# Berberine Induces Combined Cell Death in Gastrointestinal Cell Lines

**DOI:** 10.3390/ijms24076588

**Published:** 2023-04-01

**Authors:** Shiori Mori, Rina Fujiwara-Tani, Momoko Gyoten, Shota Nukaga, Rika Sasaki, Ayaka Ikemoto, Ruiko Ogata, Shingo Kishi, Kiyomu Fujii, Hiroki Kuniyasu

**Affiliations:** Department of Molecular Pathology, Nara Medical University, 840 Shijo-cho, Kashihara 634-8521, Japan; m.0310.s.h5@gmail.com (S.M.);

**Keywords:** mitochondrial complex, autophagy, apoptosis, ferroptosis, ROS

## Abstract

Berberine (BBR) is a plant alkaloid that has various biological activities. The effects of BBR on gastrointestinal cancer (GIC) have also been investigated and anti-tumor effects such as induction of cell death have been reported. However, the mechanism of BBR-induced cell death has not been fully elucidated. To this end, we investigated the effects of BBR using three GIC cell lines. Our analyses revealed that BBR inhibited cell proliferation, invasion, sphere formation, and anticancer drug resistance in all of the cell lines. BBR also induced an increase in mitochondrial superoxide, lipid peroxide and Fe^2+^ levels, decreased mitochondrial membrane potential and respiration, decreased glutathione peroxidase 4 expression and glutathione and induced Parkin/PINK1-associated mitophagy. BBR, as well as rotenone, inhibited mitochondrial complex I and enhanced complex II, which were associated with autophagy, reactive oxidative species production, and cell death. Inhibition of complex II by malonate abrogated these changes. BBR-induced cell death was partially rescued by ferrostatin-1, deferoxamine, Z-VAD-FMK, and ATG5 knockdown. Furthermore, oral administration of BBR significantly reduced tumor weight and ascites in a syngeneic mouse peritoneal metastasis model using CT26 GIC cells. These findings suggest that BBR induced a combined type of cell death via complex I inhibition and autophagy. The marked anti-tumor and anti-stemness effects are expected to be useful as a new cell death-inducing agent for the treatment of GIC.

## 1. Introduction

Gastric cancer and colorectal cancer account for 25% of cancer deaths and 28% of cancer-related morbidities in Japan [1]. GICs account for 29% of new cancer cases and about 50% of cancer-related deaths worldwide [2,3]. In Japan, the 5-year survival rate for GIC is around 70% [1], but for cases with distant metastasis, the rate is only 6.6% for gastric cancer and 18.8% for colorectal cancer [4]. The development of novel anticancer agents is important for improving disease prognosis.

The anti-tumor effects of food nutrients, especially small molecules from plant components, have been extensively investigated. For example, curcumin has been shown to exert anti-tumor and anticarcinogenic effects by regulating intestinal flora and antioxidant effects [5,6]. Furthermore, we have previously reported that pterostilbene, which is abundant in blueberries, has a suppressive effect on cancer stem cells and promotes anti-tumor effects when used in combination with sunitinib in GIC cells [7,8].

Berberine (BBR) is a plant alkaloid that has been shown to affect intestinal immunity and reduce inflammatory cytokines [9,10]. BBR has demonstrated anti-tumor effects against multiple types of cancer, such as breast, bladder, liver, and colon [11,12,13,14]. The anti-tumor effects of BBR include suppression of growth, induction of apoptosis, promotion of autophagy, and suppression of invasion, angiogenesis, epithelial-mesenchymal transition, and metastasis [15,16,17]. However, the details of BBR-induced cell death have not been fully clarified. With this perspective, the aim of this study was to explore the effects of BBR on GIC cell lines, including a focus on how this compound affects mitochondrial functions and cell death in GIC.

## 2. Results

### 2.1. Effects of BBR on Proliferation, Invasion, and Sphere Formation in GIC Cells

**Figure 1:**
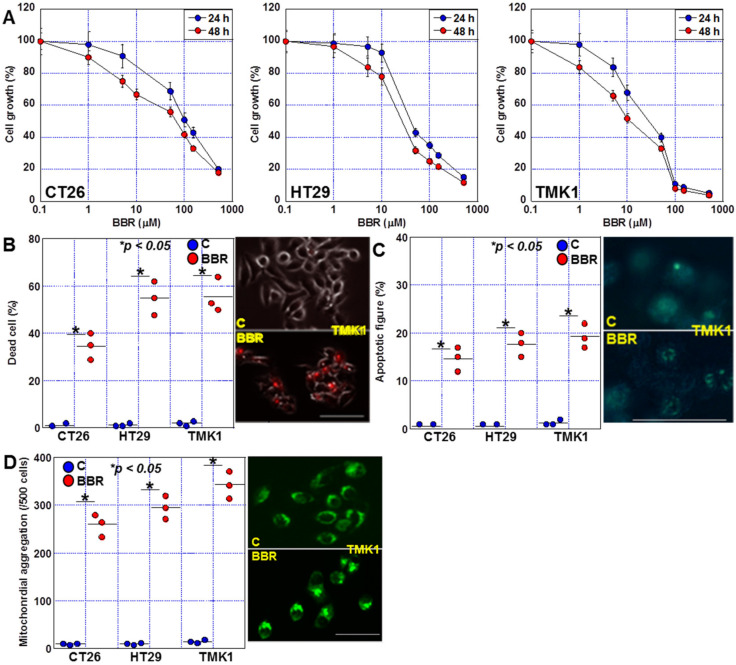
Effects of BBR on proliferation in GIC cells. (**A**) Effect of BBR on cell growth in three GIC cell lines. The cells (1 × 10^6^) were treated with BBR for 24 h and 48 h. (**B**–**D**) Effect of BBR on cell death (**B**), apoptosis (**C**), and mitochondrial aggregation (**D**) in GIC cells. Scale bar, 50 μm. BBR, berberine; GIC, gastrointestinal cancer.

To confirm the anti-tumor effects of BBR on GIC cells, we treated three GIC cell lines, CT26 (mouse colon cancer cell line), HT29 (human colon cancer cell line), and TMK-1 (human gastric cancer cell line), with various concentrations of BBR and observed concentration-dependent growth suppression of all three cell lines (Figure 1A). A time-dependent inhibition of growth by BBR was observed when 24 and 48 h treatments were compared. The IC50 value by 48 h treatment for BBR was 17.2 μM, 11.9 μM, and 9.7 μM for CT26, HT29, and TMK-1 cells, respectively.

Dead cells were confirmed by fluorescent staining in order to examine whether the suppression of cell growth was due to cell death (Figure 1B). As a result, 36%, 54% and 54% cell death were observed in CT26, HT29 and TMK1 cells, respectively. It was shown that cell growth suppression was mostly due to cell death in all three GIC cells. Next, we examined apoptotic figures and mitochondrial aggregation to examine the characteristics of cell death (Figure 1C,D). Apoptotic figures were observed in 14%, 17% and 19%, respectively, of CT26, HT29 and TMK1 cells by BBR treatment. Mitochondrial aggregation was observed in 40%, 59% and 69% of CT26, HT29 and TMK1 cells, respectively. These morphological changes suggested the possibility that BBR induces apoptosis and ferroptosis.

**Figure 2:**
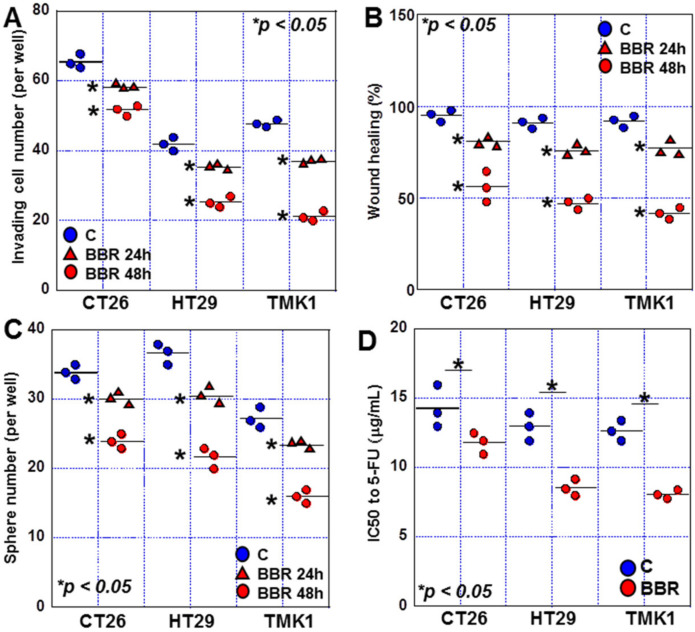
Effects of BBR on invasion, and sphere formation in GIC cells. (**A**–**D**) Effect of BBR on invasion (**A**), wound healing (**B**), sphere formation (**C**), and sensitivity to 5-FU (**D**) in GIC cells. BBR, berberine; GIC, gastrointestinal cancer; FU, fluorouracil.

Next, we examined the effect of BBR on invasive ability (Figure 2A). BBR treatment decreased invasive ability in all three GIC cell lines, with suppression rates of 25%, 40%, and 56% observed for CT26, HT29, and TMK-1 cells at 48 h, respectively. BBR treatment decreased migration ability tested by wound healing assay in all three GIC cell lines, with suppression rates of 42%, 46%, and 49% observed for CT26, HT29, and TMK-1 cells at 48 h, respectively (Figure 2B). When the effect of BBR on sphere-forming ability was examined, our analyses revealed a decrease in the sphere-forming ability of 27%, 42%, and 43% for CT26, HT29, and TMK-1 cells at 48 h, respectively (Figure 2C). Furthermore, when we examined the effect of BBR on drug sensitivity to 5-fluorouracil, our results revealed a decrease in 5-fluorouracil IC50 in all cells, with reductions of 10%, 32%, and 33% for CT26, HT29, and TMK-1 cells, respectively (Figure 2D).

Together, these data confirm that BBR inhibits GIC cell proliferation via cell death, invasion, migration, sphere formation, and drug resistance. Interestingly, the inhibitory effect of BBR appeared to be stronger in HT29 and TMK-1 cells than in CT26 cells.

### 2.2. Effects of BBR on Mitochondrial ROS Production in GIC Cells

**Figure 3:**
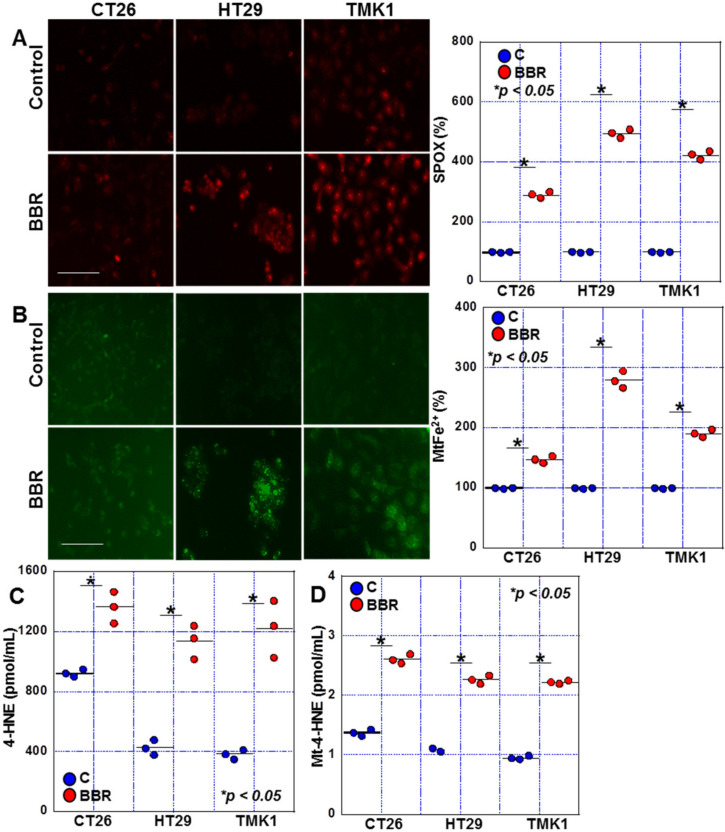
Effects of BBR on mitochondrial ROS in GIC cells. (**A**) Effect of BBR on mitochondrial superoxide (detected by mitoROS) in GIC cells. (**B**) Effect of BBR on mitochondrial Fe^2+^ (detected by mitoFerrogreen). Right panels indicate the semi-quantified values of the fluorescence intensities of the images. (**C**,**D**) Effect of BBR on 4-HNE (**C**): Whole cell lysate and (**D**): mitochondrial fraction) (detected by ELISA). Scale bar, 50 μm. Error bars represent the SD from three independent experiments. BBR, berberine; ROS, reactive oxygen species; GIC, gastrointestinal cancer; SPOX, superoxide; MtFe^2+^, mitochondrial Fe^2+^; HNE, hydroxynonenal, Mt4-HNE, mitochondrial 4-HNE.

Next, we examined the effect of BBR on mitochondrial function in GIC cells (Figure 3). When we investigated the effect of BBR (25 μM), mitochondrial superoxide levels were increased in all three GIC cell lines (Figure 3A), as did mitochondrial Fe^2+^ levels (Figure 3B). BBR also induced elevated production of 4-hydroxynonenal (HNE), a lipid peroxide, in all cell lines (Figure 3C). Moreover, BBR treatment led to an increase in mitochondrial 4-HNE levels in all cell lines (Figure 3D). In the above assays, the retention of superoxide and mitochondrial iron induced by BBR was more strongly observed in HT29 and TMK-1 cells than in CT26 cells.

### 2.3. Alterations in the Energy Metabolism of GIC Cells Treated with BBR

**Figure 4:**
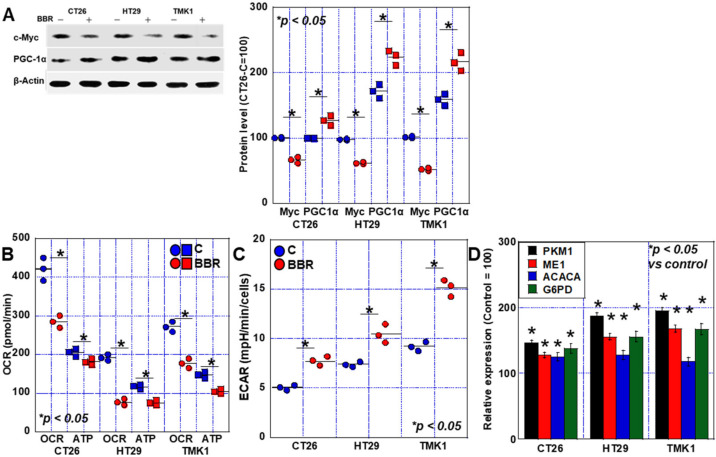
Effects of BBR on mitochondrial energy metabolism in GIC cells. (**A**) Effect of BBR on the expression of energy metabolism-related genes detected by Western blot. The right panel indicates the semi-quantified values of the signal intensities. (**B**) Effect of BBR on mitochondrial respiration (basal OCD) and ATP production detected by flux analysis. (**C**) Effect of BBR on glycolytic activity by Glycolytic stress test. (**D**) Expression of energy metabolism-associated genes. Error bars represent the SD from three independent experiments. BBR, berberine; GIC, gastrointestinal cancer; PGC, peroxisome proliferator-activated receptor-gamma coactivator; OCR, oxygen consumption rate; Basal, basal OCR; ATP, ATP production; CT26-C, value of control CT26 cells; ECAR, extracellular acidification rate; PKM1, pyruvate kinase M1; ME1, malic enzyme 1; ACACA, acetyl-CoA carboxylase α; G6PD, glucose-6-phosphate dehydrogenase.

Next, we examined the protein production of c-MYC and peroxisome proliferator-activated receptor-gamma coactivator (PGC)-1α, which are genes related to energy metabolism. Our analyses showed that the protein level of c-Myc, which is associated with glycolysis, decreased in response to BBR in all cells. In contrast, PGC-1α levels, which are associated with oxidative phosphorylation, were increased in all cells (Figure 4A). Furthermore, when mitochondrial respiration and ATP production were examined, we observed a decrease in basal oxygen consumption rate (OCR) and ATP in response to BBR in all cells (Figure 4B). However, the decrease in ATP was mild compared to the decrease in OCR.

Considering the possibility that glycolysis is involved in maintaining ATP levels, we performed a glycolytic stress test (Figure 4C). Glycolytic activity was increased in all cells by BBR treatment. Since ECAR was upregulated in spite of decreased c-myc expression, we investigated the expression of genes related to energy metabolism (Figure 4D). Expression of pyruvate kinase M (PKM, glycolysis), enzyme-1 (ME1, glutaminolysis), and acetyl-CoA carboxylase α (ACACA, citric acid cycle), glucose-6-phosphate dehydrogenase (G6PD, pentose phosphate pathway) was also enhanced in all three GIC cells by BBR treatment. 

### 2.4. Changes in Mitophagy in BBR-Treated GIC Cells

**Figure 5:**
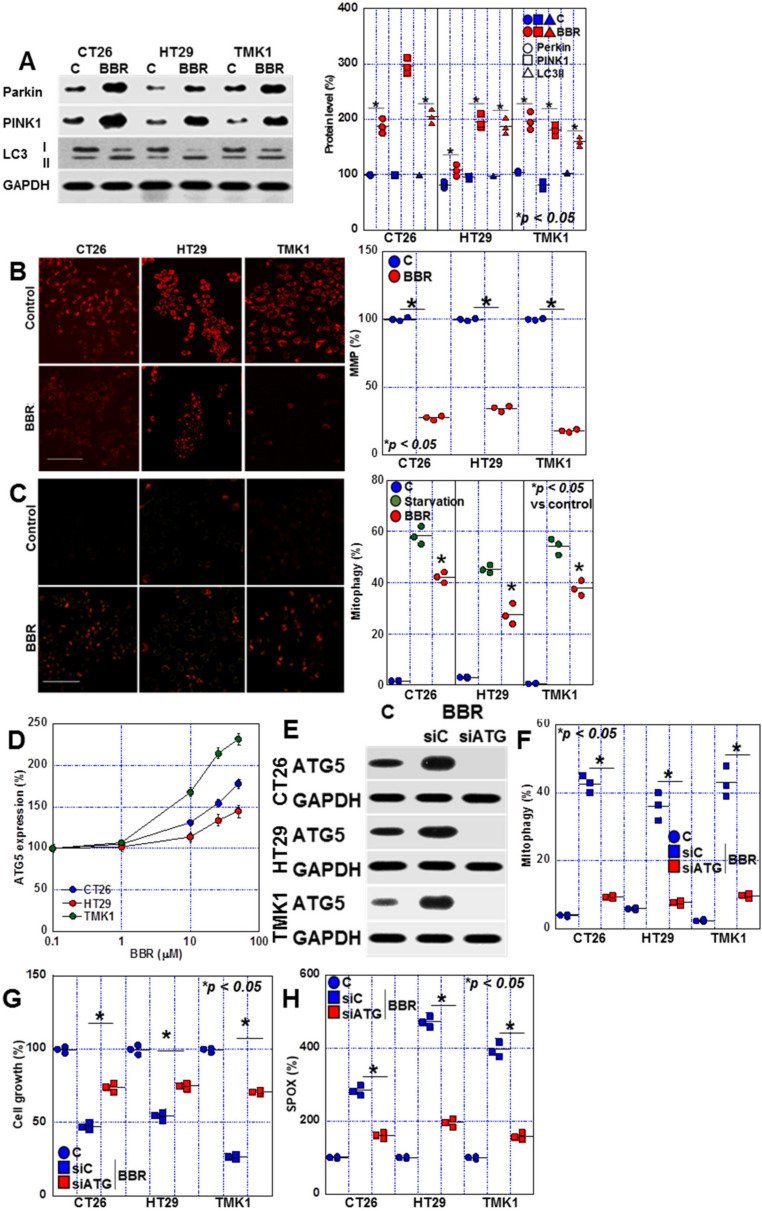
Effects of BBR on autophagy in GIC cells. (**A**) Effect of BBR on protein levels of autophagy-related genes detected by Western blot. The right panel indicates the semi-quantified values of the signal intensities. (**B**) Effect of BBR on mitochondrial membrane potential (detected by TMRE) in GIC cells. The right panels indicate the semi-quantified values of the fluorescent intensities. Scale bar, 50 μm. (**C**) Effect of BBR on mitophagy detected by Mitophagy Detection Kit. Mitophagy cell figures were counted in 200 cells. For positive control, cells were treated by serum starvation (48 h). Scale bar, 50 μm. (**D**) Effect of BBR on ATG5 protein expression. (**E**) Effect of small interference RNA for ATG5 (siATG) on protein levels of ATG5. (**F**–**H**) Effect of ATG5 knockdown on mitophagy (**F**), cell growth (**G**) and mitochondrial superoxide (SPOX) production (**H**). Error bars represent the SD from three independent experiments. BBR, berberine; GIC, gastrointestinal cancer; PINK1, PTEN induced putative kinase 1; GAPDH, glyceraldehyde 3-phosphate dehydrogenase; MMP, mitochondrial membrane potential; TMRE, tetramethylrhodamine methyl ester; ATG, autophagy-related; siC, control small interference RNA.

BBR has been reported to affect autophagy [18], and we, therefore, examined the production of autophagy-related proteins (Figure 5A). Protein levels of Parkin, PTEN induced putative kinase 1 (PINK1) were increased in all three cell lines. BBR increased LC3II and decreased LC3I. BBR also reduced mitochondrial membrane potential and induced mitophagy (Figure 5B,C). BBR treatment induction of Autophagy related 5 (AGT5) expression in a dose-dependent manner in GIC cells (Figure 5D). AGT5 knockdown abrogated BBR-induced upregulation of ATG5, mitophagy, cell growth inhibition and mitochondrial ROS production (Figure 5E–H). These findings suggest that BBR-induced mitophagy is associated with cell death via ROS production.

### 2.5. Induction of Cell Death by BBR in GIC Cells

**Figure 6:**
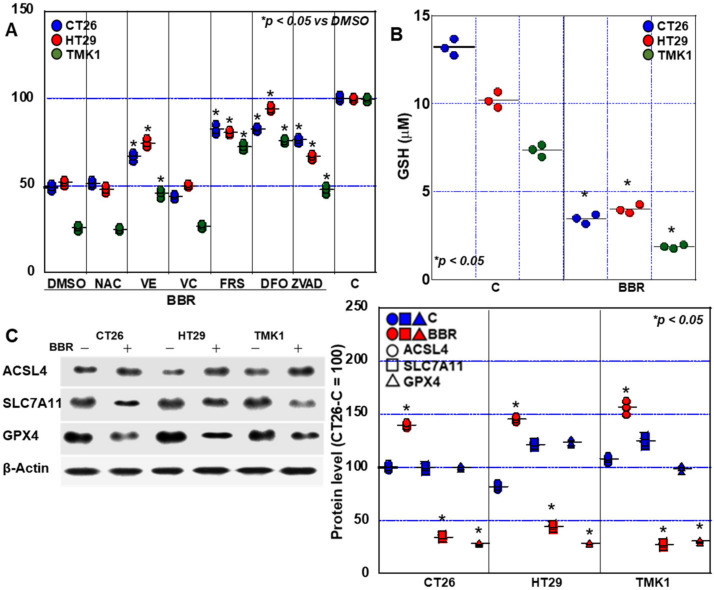
Investigation of cell death mechanisms in GIC cells. (**A**) The effect of cell death inhibitors on cell growth was examined in BBR-treated GIC cells. Cells were treated with BBR and inhibitors for 48 h. DMSO was used as a vehicle control. (**B**) Intracellular GSH concentration. (**C**) Levels of ferroptosis-associated proteins. The right panel indicates the semi-quantified values of the signal intensities. Error bars represent the SD from three independent experiments. BBR, berberine; GIC, gastrointestinal cancer; DMSO, dimethyl sulfoxide; NAC, N-acetyl-L-cysteine; VE, vitamin E; VC, vitamin C; FRS, ferrostatin-1; DFO, deferoxamine; ZVAD, Z-VAD-FMK; C, control; ACSL4, acyl-CoA synthetase long chain family member 4; SLC7A11, solute carrier family 7 member 11; GPX4, glutathione peroxidase 4; GSH, glutathione.

Next, we examined BBR-induced cell death using various inhibitors (Figure 6A): in CT26 cells, BBR-induced growth inhibition was rescued from 48% to 78% by the apoptosis inhibitor Z-VAD-FMK and from 48% to 85% and 86% by ferrostatin-1 (FRS) and deferoxamine (DFO), respectively. In HT29 cells, growth inhibition by BBR was rescued from 55% to 64% by Z-VAD-FMK and from 55% to 82% and 95% by FRS and DFO, respectively. In TMK1 cells, BBR-induced growth inhibition was rescued by Z-VAD-FMK from 28% to 45% and by FRS and DFO from 28% to 68% and 75%, respectively. To investigate the relationship between ROS and BBR-induced cell death, we also treated GIC cells with vitamin E and NAC. Cell death was partially rescued by vitamin E.

Intracellular glutathione (GSH) was decreased by BBR in all cells (Figure 6B). Next, we examined the expression of ferroptosis-related proteins (Figure 6C). Expression of acyl-CoA synthetase long-chain family member 4 (ACSL4), which provides a substrate for lipid peroxidation, was upregulated in all cell lines by BBR. In contrast, the expression of solute carrier family 7 member 11 (SLC7A11) and glutathione peroxidase 4 (GPX4), which inhibit oxidative reactions, was suppressed in all cells. Furthermore, these results indicate that BBR impairs the antioxidant mechanism in GIC cells.

### 2.6. Complex I Inhibition by BBR

**Figure 7:**
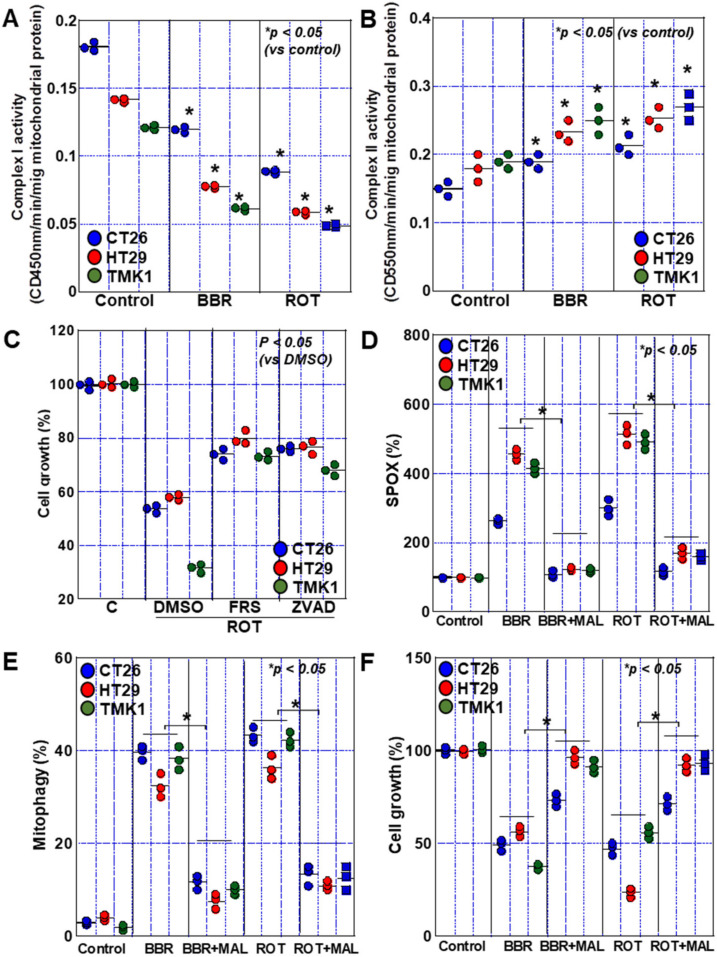
Effect of inhibition of mitochondrial complex I on cell death. (**A**,**B**) Inhibitory effect of BBR and ROT on activities of mitochondrial complex I (**A**) and II (**B**). (**C**) Effect of cell death inhibitors on rotenone-induced cell death. (**D**–**F**) Effect of malonate on mitochondrial superoxide production (**D**), mitophagy (**E**) and cell growth (**F**). Error bars represent the SD from three independent experiments. BBR, berberine; ROT, rotenone; FRS, ferrostatin-1; ZVAD, Z-VAD-FMK; DMSO, dimethyl sulfoxide; MAL, malonate; SPOX, superoxide.

Furthermore, since BBR has an inhibitory effect on complex I [19], the activities of complex I and II in BBR-treated GIC cells were examined (Figure 7A,B). BBR suppressed complex I activity, whereas complex II activity was inversely increased. When cells were treated with rotenone, the same reactions were observed. In rotenone-treated cells, growth inhibition was partially rescued by ferrostatin-1 and pan-caspase inhibitor, which resembled the effects ofBBR treatment (Figure 7C). Treatment with a complex II inhibitor, malonate, abrogated BBR- and rotenone-induced mitochondrial ROS production and mitophagy (Figure 7D,E). Subsequently, complex II inhibition rescued BBR- and rotenone-growth inhibition (Figure 7F).

### 2.7. Effect of BBR in Mouse Peritoneal Dissemination Model Using CT26 Cells

**Figure 8:**
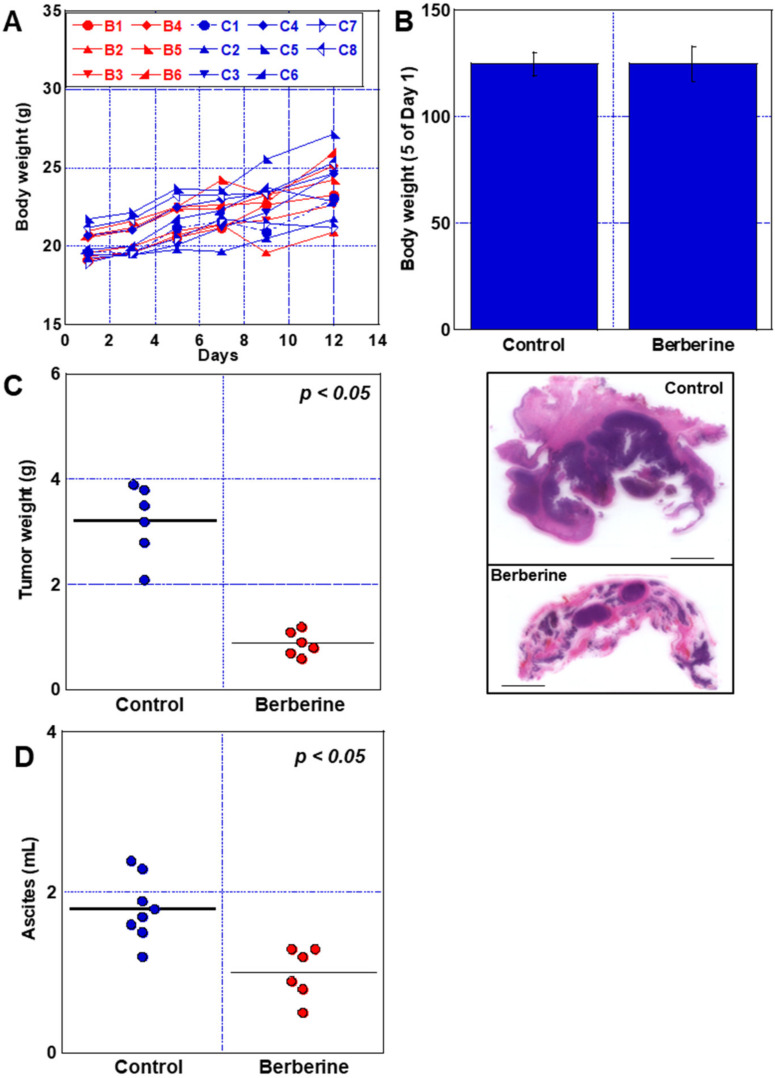
Effect of BBR in mouse peritoneal dissemination model using CT26 cells. The mice were administered with BBR (48 μg/mL in water, free drink, mean administration 15.2 mg/kg body weight/day) over the course of the experiment. (**A**) Time course of body weight of each mouse. B1–B6 indicate BBR-treated mice (6 mice). C1–C8 indicate untreated control mice (8 mice). (**B**) Body weight at Day 12 is presented as a percentage of body weight on Day 1. (**C**) Tumor weight at Day 12 and loupe imaging of diaphragmatic tumors. Scale bar, 5 mm. (**D**) Ascitic volume at Day 12. Error bars represent the SD from mice within the group. BBR, berberine.

Finally, we examined the effects of BBR in a mouse model. We used a syngeneic tumor mode to obtain data that are more extrapolatable to humans. CT26 cells were peritoneally inoculated into syngeneic BALB/c mice (Figure 8). No change in body weight was observed between the control and BBR-administered groups (Figure 8A,B). In the BBR-treated group, the mean weight of peritoneal disseminated tumors decreased to 28% of the mean tumor weight in the control group (Figure 8C). Histological examination showed that hematoxylin-positive tumors were reduced in BBR-treated mice. Furthermore, ascites was reduced by 56% in the BBR group (Figure 8D). Thus, BBR strongly suppressed peritoneal dissemination of CT26 cells.

## 3. Discussion

In this study, we demonstrated that BBR exerts an anti-tumor effect on three GIC cell lines by inducing cell death. Our results show that BBR inhibited mitochondrial complex I activity and promoted complex II activity. In mitochondria, BBR also caused iron accumulation, increased ROS production, increased oxidized lipids, impaired antioxidant system, reduced mitochondrial membrane potential, and suppressed oxidative phosphorylation. Furthermore, BBR promoted Parkin/PINK1-mediated mitophagy. Further, BBR-induced cell death was suppressed by ferroptosis inhibitors, an apoptosis inhibitor, complex II inhibition, and mitophagy inhibition.

We investigated the relationship between cell death and complex I because BBR has a complex I inhibitory effect. BBR, like rotenone, inhibited complex I activity and stimulated complex II activity. Mutual compensatory actions have been reported between complexes I and II [20]. BBR induced increased ROS production, mitophagy, and cell death in GIC, also observed on rotenone treatment. Furthermore, inhibition of complex II by malonic acid counteracted the effects of BBR and rotenone, suggesting that the activation of complex II as well as inhibition of complex I is involved in the action of BBR. Rotenone enhances ROS production by complex II [21], induces mitochondrial depolarization [22], increases mitophagy [23], and inhibits mitochondrial Fe^2+^ oxidation, which results in cytotoxicity [24]. These suggest that the mitochondrial effects of BBR, like rotenone, are based on the inhibition of complex I. 

It is known that BBR and rotenone induce apoptosis through enhanced ROS production [17,25]. In our study, although BBR-induced cell death was suppressed by a pan-caspase inhibitor, the effect was partial. The partial suppression of cell death was also observed after treatment with a ferroptosis inhibitor and an iron chelator. Ferroptosis is defined by the Nomenclature Committee on Cell Death 2018 as “A form of regulated cell death initiated by oxidative perturbations of the intracellular microenvironment that is under constitutive control by GPX4 and can be inhibited by iron chelators and lipophilic antioxidants” [26]. Ferroptosis is a cell death triggered by lipid peroxidation dependent on the generation of reactive oxygen species (ROS) and iron accumulation [16]. Our data show that BBR induces cell death in GIC cells accompanied by increased mitochondrial superoxide and 4-HNE levels, decreased SLC7A11, and impaired antioxidant mechanisms, indicated by decreased GPX4 expression and decreased GSH. Furthermore, BBR-induced cell death is partially rescued by the iron chelator DFO and the ferroptosis-inhibitor FRS, suggesting that BBR induces ferroptosis as well as apoptosis. BBR and rotenone have a common mechanism of inhibiting complex I, but differ in that BBR accumulates mitochondrial Fe^2+^, whereas rotenone decreases it [27]. Rotenone-induced apoptosis was suppressed by VC and NAC [25], but our data did not rescue BBR-induced cell death by VC or NAC. This suggests that mitochondrial ROS via accumulation of Fe^2+^ in mitochondria by BBR reduces the cell death inhibitory effect of water-soluble antioxidants.

In our study, the suppression of BBR-induced mitophagy reduced ROS production and cell death. Mitophagy is thought to dispose of mitochondria damaged from increased ROS production and contribute to the reduction of ROS [28]. Thus, autophagy contributes to cell survival. In contrast, recent studies suggest that autophagy may be a physiological cell death process that connects various cell death pathways via various molecular mediators [29]. Autophagy induces programmed cell death under some conditions [30,31] and cell death associated with organelle reduction [32]. There are reports of inhibition of autophagy due to the suppression of ATG3, ATG5, and ATG6/7 [33,34,35]. Autophagy and apoptosis act in concert in T-lymphocytic leukemia treated with arsenic trioxide [36], in imatinib treatment of Kaposi’s sarcoma [37], in the effect of vitamin K on leukemic cells [38].

Autophagy acts both to suppress and promote cell death in some cancer cells [39,40]. In our study, BBR promoted mitophagy through Parkin/PINK1-mediated pathway. There is also a report that BBR promotes autophagy by suppressing the mTOR, Akt, and MAPK pathways [41].

Autophagy enhances ROS production [42], which promotes apoptosis and ferroptosis [43,44]. Our results also show that the inhibition of autophagy by ATG5 knockdown outperforms the effects of inhibitors of apoptosis and ferroptosis alone on BBR-induced cell death. In our experiments, the inhibition of autophagy significantly suppressed ROS production, which supports the scenario that autophagy promotes apoptosis and ferroptosis by enhancing ROS production. This suggests that autophagy may play an integral role in multiple types of cell death.

Our observations showed aggregation of mitochondria by BBR. This correlates with the link between ferroptosis and promotion of mitochondrial fusion [45]. However, for detailed examination, observation of mitochondrial micromorphology should be needed. This point is important for understanding the involvement of BBR in mitoptosis. In addition, there are reports that BBR is involved in necroptosis [46] and pyroptosis [47]. These are subjects for future studies.

Our data show that BBR upregulated the expression of glycolysis-related genes and increases ECAR, despite the repression of c-Myc expression. One possibility is that glycolysis was promoted as feedback to the suppression of Complex I by BBR. c-Myc has been reported to suppress ferroptosis [48], and suppression of c-myc expression by BBR is considered to be one of the ferroptosis-promoting effects. It is suggested that FOXO3a-mediated suppression of c-Myc expression is involved in this background [49]. c-Myc promotes the expression of glycolytic genes, whereas its suppression inactivates transcriptional conversion from PKM1 to PKM2 and promotes lactate fermentation [50,51]. Increased ROS promotes the expression of the antioxidant gene ME1 [52]. These changes are suggested to be involved in the paradoxical enhancement of glycolysis by BBR.

In our study, BBR-induced GIC cells with reduced sphere-forming ability, reduced anticancer drug resistance, and metastatic potential in the mouse model. All these suggest that BBR suppresses the stemness of cancer cells. Recently, it has become evident that stem cells depend on oxidative phosphorylation for their energy metabolism [53] and require appropriate levels of oxidative stress [54]. BBR suppresses oxidative phosphorylation and induces excessive oxidative stress in GIC cells, which may result in the suppression of stemness. Cancer stem cells are thought to bring about the ultimate malignancy of cancer, such as metastasis, recurrence and treatment resistance [55,56], and its effective targeting has become an important goal in cancer therapy [57].

Our study showed that BBR induces apoptosis and ferroptosis by inhibiting mitochondrial complex I and promoting autophagy, leading to combined cell death in the GIC and suppressing stemness. Thus, the application of BBR to cancer therapy is considered to be of great value.

## 4. Materials and Methods

### 4.1. Cell Lines and Reagents

The HT29 human colorectal carcinoma cell line was purchased from Dainihon Pharmacy Co. (Tokyo, Japan). TMK-1 human gastric carcinoma cells were a gift from Professor Wataru Yasui (Molecular Pathology, Hiroshima University, Hiroshima, Japan), and the CT26 murine colon carcinoma cell line was a gift from Professor I. J. Fidler (MD Anderson Cancer Center, Houston, TX, USA). Cells were cultured in Dulbecco’s modified Eagle’s medium supplemented with 10% fetal bovine serum at 37 °C in 5% CO_2_. 

BBR (25 μM for 48 h unless otherwise specified, Tokyo Chemical Industry Co., Ltd., Tokyo, Japan), 5-fluorouracil (5-FU), N-acetyl-L-cysteine (NAC 1 mM), rotenone (0.5 μM) (Sigma-Aldrich Inc., St. Louis, MO, USA), malonate (0.5 mM), vitamin E (20 μM) and vitamin C (1000 μM) (Fujifilm-WAKO Chemicals, Osaka, Japan), Z-VAD-FMK (ZVAD, 20 μM) (Santa Cruz Biotechnology, Santa Cruz, CA, USA), ferrostatin-1 (FRS, 2 μM), deferoxamine (DFO, 200 μM) (Cayman Chemicals, Ann Arbor, MI, USA), were purchased from the manufacturers listed. 

Cell death was detected using Live-or-Dye™ Fixable Viability staining kit (Cosmobio, Tokyo, Japan). Dead cells were counted by observation of 500 cells. Apoptotic figures were detected by Hoechst 33342 dye (Sigma) apoptotic figures were counted by observation of 500 cells.

### 4.2. Cell Growth

Cell growth was assessed using the 3-(4,5-dimethylthiazol-2-yl)-5-(3-carboxymethoxyphenyl) -2-(4-sulfophenyl)-2H-tetrazolium (MTS)-based Celltiter 96 Aqueous One Solution Cell Proliferation Assay kit (Promega Corporation, Madison, WI, USA), as previously described [8]. The absorbance in each well was measured using a multiscan FC microplate photometer at a wavelength of 490 nm. 

### 4.3. Chamber Invasion Assay

A modified Boyden chamber assay was performed to examine the in vitro invasive ability of the GIC cells [58]. Following incubation at 37 °C for 24 h, the filters were carefully removed from the inserts, stained with hematoxylin for 10 min, and mounted on microscope slides. The number of stained cells in each insert was counted at 100× magnification. Invasive activity was quantified by calculating the average number of cells per insert well. These experiments were performed in triplicate.

### 4.4. Wound Healing Assay

“Wound” was created by a straight-line scratch across the GIC cells forming confluent monolayer. The average width of the wound was measured, and its narrowing was calculated [58].

### 4.5. Sphere Assay

For this assay, 1000 cells per well were seeded on uncoated bacteriological 35 mm dishes (Coning Inc., Coning, NY, USA) in 3D Tumorsphere Medium XF (Sigma), and cultured with or without BBR (25 μM). After 7 days, digital images of the spheres were captured using a BZ-X710 All-in-One fluorescence microscope (KEYENCE, Osaka, Japan) and sphere sizes were measured using NIH ImageJ software (version 1.52, Bethesda, MD, USA). 

### 4.6. Mitochondrial Imaging

Mitochondrial functions were examined using fluorescent probes. After treatment with or without BBR (25 μM), cells were incubated with the probes for 30 min at 37 °C and then photographed using an All-in-One fluorescence microscope (KEYENCE). We used MitoROS (mitochondrial superoxide) (10 μM, AAT Bioquest Inc., Sunnyvale, CA, USA) to assess oxidative stress, mitoGreen (100 nM, PromoCell GmbH, Heidelberg, Germany) to assess mitochondrial volume, tetramethylrhodamine ethyl ester (TMRE) (200 nM, Sigma-Aldrich) to assess MMP, and mitoFerrogreen (20 nM, Dojindo, Kumamoto, Japan) to assess mitochondrial iron (Fe^2+^). Mitophagy was detected using a Mitophagy Detection Kit (Dojindo), according to the manufacturer’s instructions.

### 4.7. Enzyme-Linked Immunosorbent Assay (ELISA) and Fluorometric Assay

Whole-cell lysates and mitochondrial fraction were prepared, as previously described using RIPA buffer containing 0.1% SDS (Thermo Fisher Scientific, Tokyo, Japan) [51] and mitochondria isolation kit for cultured cells (Thermo Fisher Scientific), respectively. Protein assays were performed using a Protein Assay Rapid Kit (Wako Pure Chemical Corporation, Osaka, Japan). By using the extracted proteins, an ELISA kit was used to measure the concentration of 4-hydroxynonenal (HNE) (Abcam, Cambridge, MA, USA), GSH (Biomatik, Kitchener, ON, Canada), human ATG5 (Life Span Biosciences, Seattle, WA, USA), and mouse ATG5 (Bioassay Technology Laboratory, Shanghai, China) in whole-cell lysates, according to the manufacturer’s instructions.

### 4.8. Extracellular Flux Analysis

To analyze mitochondrial respiration and ATP production, we used a Seahorse XF Analyzer (Agilent Technologies, Santa Clara, CA, USA), which measures extracellular flux in live cells. The cells were collected immediately after treatment, transferred into the wells of an XF plate at densities of 2 × 10^4^ cells/well, and incubated overnight. The following day, the medium in the XF plate was replaced with XF DMEM medium 1 h prior to the assay, and a Mito Stress Test (Seahorse XF Cell Mito Stress Test, Agilent) was performed according to the manufacturer’s protocol. The oxygen consumption rate (OCR) was measured under the following conditions: 2 µM (CT26 and HT29) or 3 µM (TMK-1) oligomycin, 0.5 µM carbonyl cyanide-p-trifluoromethoxyphenylhydrazone, and 0.5 µM rotenone/antimycin A. The OCR was normalized to the total cellular protein concentration, which was determined after protein extraction from the analyzed cells.

### 4.9. Glycolytic Stress Test

The extracellular acidification rate (ECAR) of GIC cells was measured using a modified glycolytic stress test in the Seahorse XFe24 Extracellular Flux Analyzer with Seahorse XF24 FluxPaks (Agilent Technologies, Santa Clara, CA, USA). GIC cells were cultured in a growth medium in 6-well plates with the ascites or the cultured medium before Seahorse experiments. GIC cells (1 × 10^4^ cells/well) were later plated in the XF base medium (Agilent Technologies) containing 200 mM L-glutamine and 5 mM HEPES, as recommended by the manufacturer for glycolytic assays. The sensor cartridge apparatus was rehydrated one day in advance by adding 1-mL XF Calibrant to each well and incubating at 37 °C until needed. The injection ports of the sensor cartridge apparatus were loaded with the following drugs, in chronological order of four injections, to meet the indicated final concentrations in the wells: 10 mM glucose, 1 µM oligomycin, 1 µM rotenone, and 5 µM antimycin A (combined injection), and 50 mM 2-deoxyglucose. Treatment with the rotenone/antimycin combination allowed assessment of the impact of electron transport on ECAR by respiratory acidification coupled to passage of some glycolytic pyruvate through the TCA cycle to supply respiration.

### 4.10. qRT-PCR for RNA

Total cellular RNA was isolated from primary OSCC tissues using TRIzol reagent (Invitrogen) and reverse-transcribed using the Prime Script RT reagent kit together with gDNA Eraser (Perfect Real Time; Takara, Kyoto, Japan) in accordance with the manufacturer’s instructions. RNA expression was analyzed using qRT-PCR, with reactions performed in triplicate using a SYBR Green PCR kit (Takara). Glyceraldehyde3-phosphate dehydrogenase (GAPDH) mRNA was used as the internal control. The primer sets were listed in Table 1.

### 4.11. Animals

Five-week-old male BALB/c mice were purchased from SLC Japan (Shizuoka, Japan). The animals were maintained in a pathogen-free animal facility under a 12 h light/dark cycle in a temperature (22 °C)- and humidity-controlled environment, in accordance with the institutional guidelines approved by the Committee for Animal Experimentation of Nara Medical University, Kashihara, Japan, following the current regulations and standards of the Japanese Ministry of Health, Labor and Welfare (approval no. 12924, 5 November 2020). Animals were acclimated to their housing for seven days before the start of the experiment. For the peritoneal dissemination tumor model, CT26 cancer cells (1 × 10^7^ in 0.2 mL per mouse) were injected into the mouse peritoneal cavity. To measure tumor weight, mice were euthanized on Day 12 and the tumors were excised, while the peritoneal tumors were dissected from the intestine, mesenterium, diaphragm, and abdominal wall, with gross removal of non-tumor tissues. The largest tumor was formed on the diaphragm, and paraffin-embedded sections of the excised diaphragmatic tumor were prepared and stained with hematoxylin-eosin. BBR was diluted with distilled water to produce a final concentration of 48 mg/mL. The solutions were ultrasonically treated for 1 h, and fully vortexed for 30 min. BBR solution was administered by free drinking. The intake calculated from the amount of water consumed was 15.2 mg/kg body weight/day.

### 4.12. Western Blot Analysis

To prepare whole-cell lysates, the cells were washed twice with cold PBS and harvested. The cells were lysed with 0.1% NP-40-added RIPA buffer (Thermo Fisher) [51]. Protein assays were performed using the Protein Assay Rapid Kit (Wako). Protein lysates (25 μg) were separated on 12.5% sodium dodecyl sulfate-polyacrylamide gels, followed by electrotransfer onto a nitrocellulose filter. The membranes were incubated with primary antibodies and then with peroxidase-conjugated IgG antibodies (Agilent Technologies, Santa Clara, CA, USA). Immune complexes were detected using an ECL Western blot detection system (Amersham, Aylesbury, UK). The following primary antibodies were used, at a working dilution of 1:1000, for immunoblot analyses: antibodies against c-Myc (#18583), PGC-1α (#4259), SLC7A11 (#98051), Parkin (#2132), PINK1 (#6946), LC3A/B (#4108), ATG5 (#12994) (Cell Signaling Technology Japan, Tokyo, Japan), ACSL4 (bs-13129R), GPX4 (bs-3884R) (Bios Antibodies, Woburn, MA, USA), and β-actin (ab178787), GAPDH (ab8245, Abcam).

### 4.13. Small Interfering RNA

siRNAs targeting human *ATG5* were synthesized by Sigma. The following siRNA sequences were used to target the RNAs: human *ATG5*#1: 5′-CCT TTG GCC TAA GAA GAA A-3′ and *ATG5*#2: 5′-CAT CTG AGC TAC CCG GAT A-3′. siRNAs targeting mouse *atg5* were purchased from Santa Cruz (Q99J83). AllStars Negative Control siRNA was used as the control (Qiagen, Valencia, CA, USA). The cells were transfected with mixture of two siRNAs (10 nM) using Lipofectamine 3000 (Thermo Fisher Scientific) according to the manufacturer’s recommendations.

### 4.14. Activity of Mitochondrial Complex

The activities of mitochondrial complexes I and II were measured by MitoCheck Complex I and II activity assay kit (Cayman Chemical, Ann Arbor, MI, USA), respectively. The assay was performed according to the manufacturer’s instructions.

### 4.15. Statistical Analysis

Statistical significance was calculated using a two-tailed Fisher’s exact test, an ordinary ANOVA, and InStat software ver. 3.10 (GraphPad, Los Angeles, CA, USA). A two-sided *p* value of <0.05 was considered to indicate statistical significance.

## Figures and Tables

**Table 1:** Primer sets.

Gene	Gene ID	Species	Forward	Reverse
*PKM1*	NM_001411081.1	Human	GGAGAAACAGCCAAAGGGGA (EX9)	ACCCGGAGGTCCACGTCCTC (EX11) [50]
*pkm1*	NM_001253883.2	Mouse	TGTTCCACCGTCTGCTGTTT (EX9)	ACACGAAGGTCGACATCCTC (EX11)
*ME1*	NM_002395.6	Human	CCCTAGGGATTGCACACCTG	AGGAGGATAAAGCCGACCCT
*me1*	NM_001198933.1	Mouse	GGAGTTGCTGCAATTGGTGG	TGCAGGCCACGGATAACAAT
*ACACA*	NM_198836.3	Human	GGAGGAGGAGGGAAGGGAAT	CGAGCAGCAATAACATGGCC
*acaca*	NM_133360.3	Mouse	TTGCCATGGGGATCCCTCTA	GCTGTTCCTCAGGCTCACAT
*G6PD*	NM_001042351.3	Human	CTACCGCATCGACCACTACC	ACTGCTGGTGGAAGATGTCG
*g6pd*	X53617.1	Mouse	ATGGCAGAGCAGGTGGCC	AATATGTGTGTATCAGCTTGGTGG

## Data Availability

Not applicable.

## Abbreviations

BBR, berberine; GIC, gastrointestinal cancer; FRS, ferrostatin-1; DFO, deferoxamine; PINK1, PTEN induced putative kinase 1; ATG3, autophagy related 3; OCR, oxygen consumption rate; ROS, reactive oxygen species; MMP, mitochondrial membrane potential; PGC, peroxisome proliferator-activated receptor-gamma coactivator.
